# Positive Impact of Inpatient Respiratory Rehabilitation in a Rare Case of Acute Necrotizing Encephalopathy

**DOI:** 10.7759/cureus.30408

**Published:** 2022-10-17

**Authors:** Sakshi Palkrit, Pallavi Bhakaney, Ruhi Kumbhare, Vishnu Vardhan

**Affiliations:** 1 Physical Therapy, Datta Meghe Institute of Medical Sciences, Wardha, IND; 2 Cardiorespiratory Physiotherapy, Datta Meghe Institute of Medical Sciences, Wardha, IND; 3 Cardiorespiratory Physiotherapy, Ravi Nair Physiotherapy College, Datta Meghe Institute of Medical Sciences, Wardha, IND

**Keywords:** bronchial hygiene, respiratory rehabilitation, physiotherapy, acute respiratory failure, acute necrotizing encephalopathy

## Abstract

Acute necrotizing encephalopathy, also known as acute necrotizing encephalopathy of childhood (ANEC) is a rare disorder characterized by respiratory or gastrointestinal infections, high fever, and rapid changes in consciousness and seizures. ANEC is a rare form of encephalopathy characterized by multiple bilateral brain lesions, primarily involving the thalami and putamina internal and external capsules, cerebellar white matter, and the brainstem segmentum. Here we present, a rare case of acute necrotizing encephalopathy in a pediatric patient, a 13-year-old boy, who was admitted with acute onset of fever. The fever was intermittent and high grade along with chills, rigor, and respiratory distress five days back. The chest x-ray showed heterogeneous opacities in bilateral lung fields. Thalami, brainstem, cerebellum, and white matter have all been shown to have a symmetric lesion in this disease on magnetic resonance imaging (MRI). Ventilation, immunoglobulin, and other supporting measures, as well as respiratory rehabilitation, were used to treat him. In pediatric intensive care units (PICU), physiotherapy is considered an important aspect of patient care. Respiratory rehabilitation included patients and their family member's counseling, airway clearance techniques, energy conservation methods, and adaptation to complex positions with the maintenance of oxygen saturation (SpO_2_). We conclude Respiratory rehabilitation with efficient family counseling is effective in the overall improvement of the patient’s condition with acute respiratory failure in acute necrotizing encephalopathy.

## Introduction

Acute necrotizing encephalopathy is a disorder marked by a respiratory or gastrointestinal illness, high fever, and fast changes in consciousness and seizures [1]. It is a rare but unique kind of acute encephalopathy with a worldwide prevalence. Numerous cases have been documented in Asia as well as in many Western nations. The majority of instances are sporadic; however, a few incidences being occasional and/or family episodes have been described, which indicates a hereditary pattern [2]. It is widely thought to be a para infectious condition caused mainly by viral infections [3]. This encephalopathy is distinguished by multifocal, symmetric brain lesions affecting the bilateral thalamus, brainstem tegmentum, cerebral periventricular white matter, and cerebellar medulla, which may be detected on computed tomography and magnetic resonance imaging (MRI) [4]. In certain cases, a viral origin has been hypothesized; hence, a viral prodromal may precede neurological abnormalities. However, both environmental and host variables may be implicated in the causative link between viral infections and ANE, as well as the precise pathophysiology of ANE. Only around 10% of the patients make a complete recovery. There were reversible imaging alterations in cases that had a favorable result [5]. In such situations, critically ill patients present significant diagnostic challenges [6].

ANE has no distinct clinical symptoms or indications, and the exact pathophysiology and risk factors are unknown. The exact cause of acute necrotizing encephalopathy of childhood (ANEC) and the pathogens that cause it are unknown; however, mycoplasma, influenza virus, herpes simplex virus, and human herpes virus-6 are among the most prevalent diseases that worsen the condition. It is thought to be caused by a cytokine storm [7]. Tumor necrotizing factors and interleukins 1 and 6 are the cytokines that can speed up the disease. The autosomal-dominant types of ANE, both recurrent and familial, were discovered to be incompletely autosomal-dominant. Missense mutations in the gene that encodes the nuclear pore protein Ran binding protein 2 (RANBP2) were also discovered [8]. Through this case report, the rationale was to provide insight into the role of a physiotherapist in the acute necrotizing encephalopathy of a 13-year-old boy admitted to the PICU.

## Case presentation

A previously healthy 13-year-old boy with no prior medical history was referred to a rural hospital on November 7, 2021. As narrated by the father, the patient had an acute onset of fever, which was intermittent and high-grade, along with chills, rigor, and difficulty in breathing. Immediately he was taken to a local hospital where medications were given, and the fever was managed. After two days, there was an episode of fever, followed by two episodes of vomiting as well as loose stools. He developed an altered sensorium the same evening. There were no witnessed convulsions or abnormal movements at home or en route to the hospital. The patient had mild intermittent asthma which was managed by albuterol nebulization. Due to desaturation, he was given 8 liters of O_2_ support to maintain saturation and was shifted to the PICU. Necessary investigations like Complete blood count, kidney function test, liver function test, blood culture, arterial blood gas, an x-ray of the chest, and MRI of the brain were done. The patient received ceftriaxone and acyclovir along with vitamin K and adrenaline. He was also given fludrocortisone and immunoglobulins.

**Figure 1:**
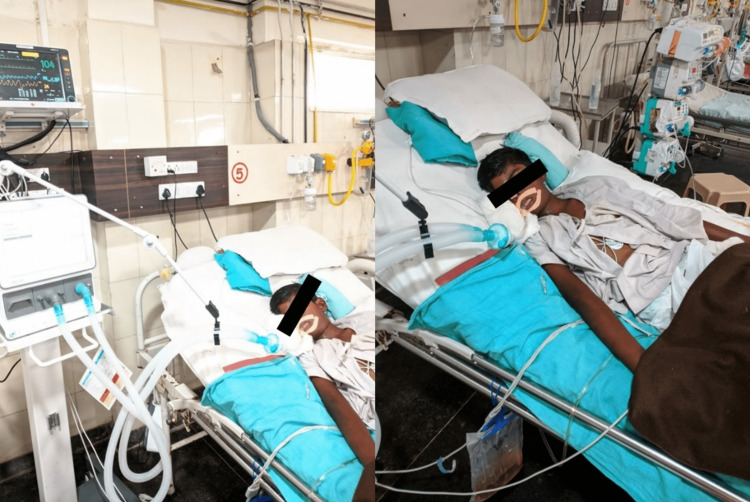
The patient on the day of assessment on a ventilator.

He presented with a poor Glasgow coma scale (GCS) with a score of 6/15 associated with an episode of desaturation, for which he was intubated with an endotracheal tube for one week. He was diagnosed with acute necrotizing encephalopathy on November 17, 2021. Given prolonged intubation for nine days, a tracheostomy was done, and he was put on a mechanical ventilator on the synchronized intermittent mandatory ventilation + pressure regulated volume control (SIMV + PRVC) mode shown in Figure 1. The chest x-ray showed heterogeneous opacities in bilateral lung fields with decreased air entry in lower zones for which a physiotherapy referral was given. Pediatric respiratory rehabilitation was started for him on the fourth^ ^day after admission. After two weeks, his condition improved, and he was put on the synchronized intermittent mandatory ventilation + pressure support (SIMV+PS) mode of the ventilator. After a week, he was put on pressure support/continuous positive airway pressure (PS + CPAP) mode of the ventilator for eight days. The weaning trials of the ventilator were started. He was able to maintain spontaneous breathing for 48 hours and then he was decannulated. After decannulation, he was moved to the pediatric ward and oral feeding was started for him.

Clinical findings

On examination, the GCS of the patient was six. On inspection, the patient was assessed in a lying position on the bed in a semi-fowler’s position. The chest was bilaterally symmetrical, and no deformity was observed. The patient was using the Accessory muscles for respiration. The patient was on the mechanical ventilator in SIMV+PRVC mode, Positive end-expiratory (PEEP) was 5 cm H_2_O, tidal volume was 420 mL, and Fraction of inspired oxygen (FiO_2_) was 80%. The Foley catheter, Ryle’s tube, and the central venous line were present in situ. The tone and Manual muscle testing findings are mentioned in Table 1. On observation, the heart rate was 82 beats per minute with a regular rhythm. The blood pressure was 116/84 mmHg and SPO2 was 98%. The respiratory rate was 30 breaths per minute, regular in rhythm, and of abdominal type. Chest excursion was bilaterally symmetrical but reduced at the supra-mammary, mammary, and infra-mammary levels. The chest expansion was reduced at all three levels, i.e., axillary level, nipple level, and xiphisternum level. Coarse crackles were heard in the bilateral basal segment on auscultation.

**Table 1:** Findings of the tone and manual muscle testing in the upper and lower limb. MMT-Manual muscle testing

Tone:	Right	Left
Upper limb	Normal	Normal
Lower limb	Normal	Normal
MMT		
Upper limb	2+	2+
Lower limb	2+	2+

Diagnostic assessment

**Figure 2:**
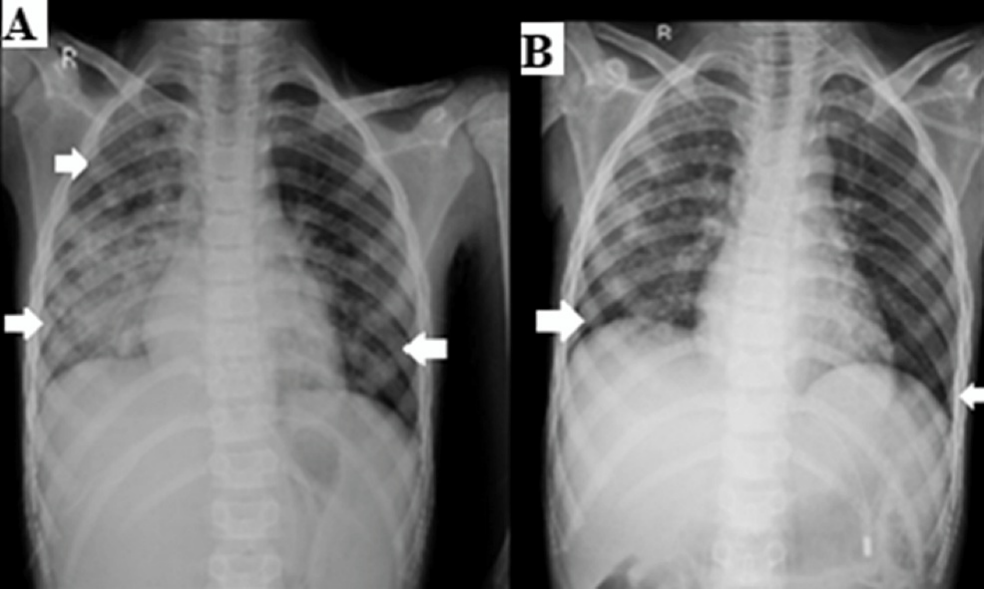
(A) X-ray of the chest shows consolidation in bilateral lower lobes and infiltrates seen in upper and middle lobes indicating an infective etiology. (B) Chest x-ray of the chest after two months of respiratory rehabilitation.

Laboratory investigations included microbiology and culture reports, which stated the growth of Pseudomonas aeruginosa and Klebsiella pneumonia. The radiological investigations included chest x-ray showing consolidation in bilateral lower lobes and infiltrates seen in upper and middle lobes (Figures 2A, 2B) and MRI showing bilateral almost symmetrical altered signal intensity areas with surrounding edema in bilateral thalami, lentiform nucleus, midbrain, and pons appearing hyperintense on T2WI/FLAIR, hypointense on T1WI, showing no post-contrast enhancement, with areas of restriction on DWI and corresponding low signal on ADC, few areas showing blooming on GRE in bilateral thalami.

Therapeutic intervention

**Figure 3:**
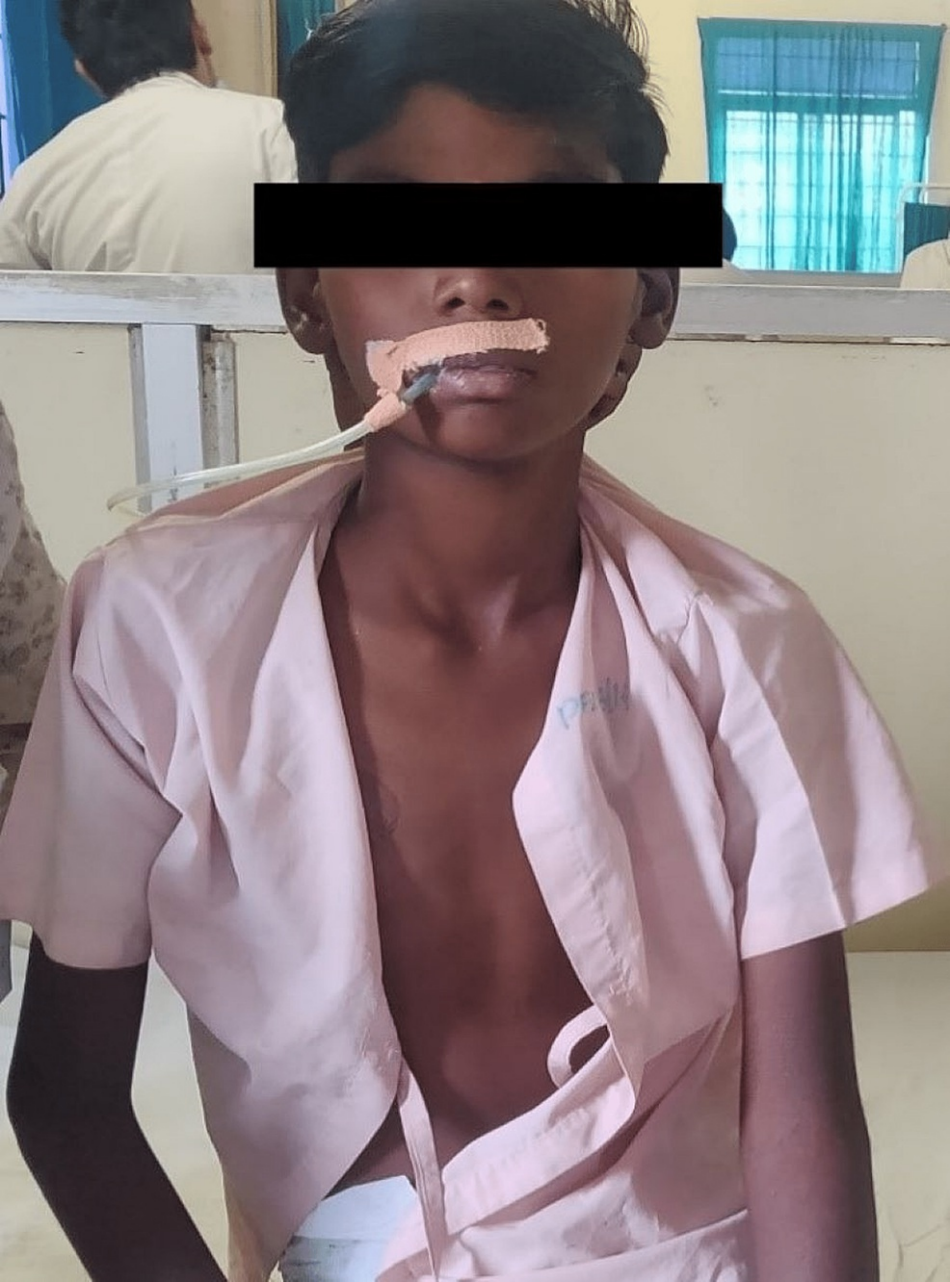
The patient at bedside sitting.

The rehabilitation protocol was of eight weeks, which was divided into weeks 1-2, weeks 3-4, weeks 5-6, and weeks 7-8 (Table 2). To enhance the patient’s functional activities, started with bedside sitting. Initially, it was for three minutes and then progressed to an increase of one minute every day (Figure 3).

**Table 2:** Respiratory rehabilitation protocol given to the patient.

Protocol			
WEEK 1- 2			
Sr. No.	Physiotherapy Treatment Goals	Therapeutic Intervention	Treatment Regimen
1	To make the patient and his family members aware of the problem, and to get their cooperation and agreement	The caregiver of the patient was educated about the patient’s condition and the importance of the physiotherapist’s role in his condition.	The caregiver of the patient was educated about the importance of the physiotherapist’s role in his condition.
2	To improve the patient’s bronchial hygiene	Manual chest percussion and chest vibrations were given for the removal of accumulated secretions followed by suctioning	Given once a day.
3	To avoid the accumulation of secretions due to long periods of immobilization	Manual positioning: Rolling	Every two-hourly positioning was given
4	To enhance chest movements, deep breathing, and expansion of lungs.	Chest proprioceptive Neuromuscular Facilitation * *(PNF): Intercostal stretch and Perioral pressure	Seven repetitions each
5	To improve the air entry into the lungs	Passive end-expiratory pressures	Seven repetitions
6	To prevent circulatory complications and muscle weakness, deformity, stiffness, and to maintain the integrity of joint.	Passive range of motion exercises and ankle toe pumps	One set consisting of 10 repetitions
WEEK 3-4			
1	To enhance airway clearance	Mechanical vibrator machine followed by suctioning	In the lower and upper lobes, twice a day
2	To prevent the secretions to accumulate	Manual positioning: rolling	Every two hour
3	To expand the epigastric movement	Chest proprioceptive neromuscular facilitation (PNF): perioral pressure	A set of seven repetitions
4	To prevent circulatory complications, stiffness, deformity	Passive range of motion exercises and ankle toe pumps	One set consisting of 10 repetitions
5	To improve the air entry into the lungs	Passive end-expiratory pressures	Seven repetitions
WEEK 5-6			
1	To improve the patient’s bronchial hygiene	Manual chest percussion and chest vibrations were given for the removal of accumulated secretions followed by suctioning	Given once a day
2	To enhance the breathing	Diaphragmatic breathing	A set of 10 repetitions
3	To maintain the integrity of joints	Active assisted exercise	A set of 10 repetitions
WEEK 7-8			
1	To enhance the health of the lungs	Diaphragmatic exercise, pursed-lip breathing exercise, and segmental breathing exercise	A set of 10 repetitions
2	To improve the mobility and expansion of the thorax	Thoracic expansion exercise	A set of 10 repetitions
3	To clear the excess secretions accumulated	Active cycle breathing technique	Three phases with five repetitions
4	To enhance patient’s functional activities	Bedside sitting	Initially for three minutes, progressed to an increase of one minute every day

Follow-up and outcomes

Follow-up of the patient was taken on the first day of the rehabilitation, Week 3, Week 6, and Week 4 by using the Glasgow Coma Scale and the Chelsea Critical Care Physical Assessment Tool (Table 3).

**Table 3:** Follow-up details of the patient

SCALES	DAY 1	WEEK 3	WEEK 6	WEEK 8
Glasgow Coma Scale	6/15	8/15	9/15	11/15
Chelsea Critical Care Physical Assessment Tool	3/50	5/50	8/50	18/50

## Discussion

Acute necrotizing encephalopathy is a crucial pediatric emergency linked to neurological morbidity, severe respiratory illness, and death [9]. It is a disorder marked by a respiratory or gastrointestinal illness, high fever, and fast changes in consciousness and seizures, and is a rare but unique kind of acute encephalopathy with a worldwide prevalence [1]. The brain is involved on a global level. This condition primarily affects children under five, and there is no gender predilection. Since the first instance was found by the Japanese researcher Mizuguchi in 1995, cases have been recorded all across the world, with more cases in Asia [4]. Patients become fast comatose and may experience convulsions. It is widely thought to be a para-infectious condition caused chiefly by viral infections [3]. ANE has no distinct clinical symptoms or indications, and the exact pathophysiology and risk factors are unknown.

The sickness reaches a peak in a few days. High fever lasts two to five days, and consciousness usually recovers after six to 10 days [10]. The prognosis for ANE usually is poor, although this has lately improved. Patients who have good outcomes may have a “mild” type of ANE, as reported by Yoshizawa et al. [11].

A well-planned interdisciplinary approach was followed for the patient. Family education plays a great role. Medical treatment includes various combinations of pharmacological agents to treat the root cause as well as to subside the current symptoms of the patient. It also prevents further deterioration of the patient’s condition by providing them with appropriate external mechanical support, majorly referring to the mechanical ventilation provided in this complicated case of acute respiratory failure.

In respiratory rehabilitation, proper lung health and bronchial hygiene were maintained by the therapeutic intervention like Manual chest percussion and vibration, use of a mechanical vibrator, chest proprioceptive neuromuscular facilitation, manual positioning, and passive end-expiratory pressure and later breathing exercises. In this case, the patient was in ICU and on a mechanical ventilator. Due to well-programmed intervention, the patient was kept more active in terms of mobility of all the joints and therefore the secondary complications were minimized. A weekly follow-up of the respiratory rehabilitation program for the patient’s current health status was planned and well-executed which helped in improving the patient’s condition and ultimately improving their quality of life.

## Conclusions

Acute necrotizing encephalopathy is a rare disorder. Respiratory rehabilitation as a form of Physiotherapy is an integral part of the recovery and management of acute respiratory failure in a case of Acute Necrotizing Encephalopathy. The benefits of respiratory rehabilitation have shown, a significant improvement in patient's lung health and well-being. This case study presents a comprehensive approach for the case of acute necrotizing encephalopathy with acute respiratory failure rehabilitation. After eight weeks of rigorous respiratory rehabilitation, the majority of the therapeutic objectives were met, including better bronchial hygiene, improved breathing pattern, increased functional vital capacity, and improved chest expansion of the patient.
